# Challenges in Liquid-Phase Exfoliation of Non-van der Waals Cr_2_S_3_

**DOI:** 10.1021/acsomega.4c02452

**Published:** 2024-11-15

**Authors:** Svetlana V. Saikova, Aleksandr Yu. Pavlikov, Diana I. Nemkova, Alexandr S. Samoilo, Denis V. Karpov, Anton A. Karacharov, Svetlana A. Novikova, Timur Yu. Ivanenko, Mikhail N. Volochaev, Galina M. Zeer, Ye Zhang, Yuri L. Mikhlin, Hans Ågren, Artem V. Kuklin

**Affiliations:** † School of Non-Ferrous Metals, Siberian Federal University, 660041 Krasnoyarsk, Russia; ‡ Institute of Chemistry and Chemical Technology, Federal Research Center “Krasnoyarsk Science Center of the Siberian Branch of the Russian Academy of Sciences”, Akademgorodok, 660036 Krasnoyarsk, Russia; § Kirensky Institute of Physics, Federal Research Center “Krasnoyarsk Science Center of the Siberian Branch of the Russian Academy of Sciences”, Akademgorodok, 660036 Krasnoyarsk, Russia; ∥ Laboratory of Electron Microscopy, Siberian Federal University, Krasnoyarsk 660041, Russia; ⊥ School of Chemistry and Chemical Engineering, University of South China, Hengyang 421001, China; # Department of Chemistry, Bar-Ilan University, Ramat Gan 52900, Israel; ∇ Department of Physics and Astronomy, Uppsala University, P.O. Box 516, SE-751 20 Uppsala, Sweden

## Abstract

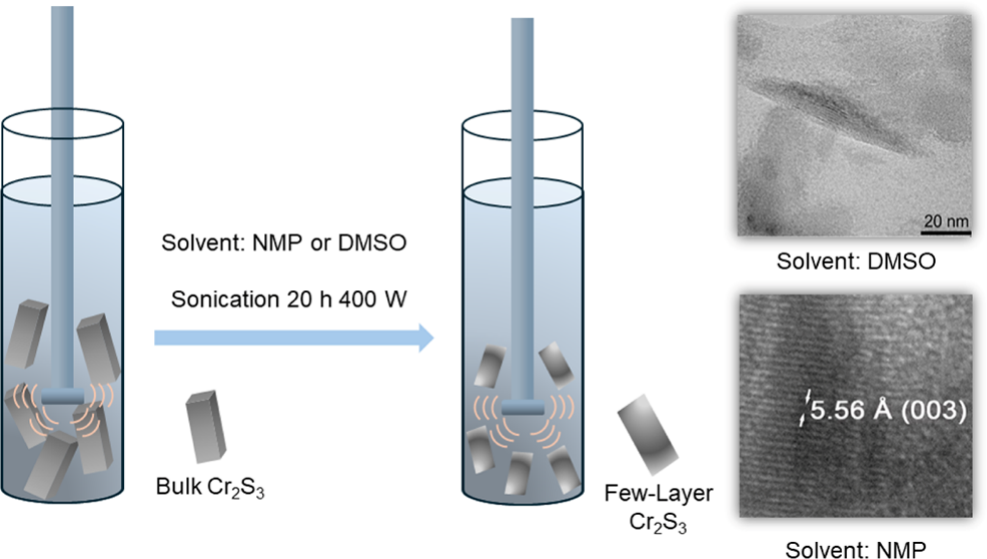

Two-dimensional (2D) chromium(III) sulfide has recently attracted increased attention from researchers due to its interesting electronic and magnetic properties and has great potential for application in spintronics and optoelectronics to create sensitive photodetectors. However, the synthesis of 2D Cr_2_S_3_ crystals is still a challenging task. At present, the mainly used method is vapor deposition, which is a poorly scalable, time-consuming, and expensive process. In this study, liquid-phase exfoliation of bulk chromium sulfide in different solvents (dimethyl sulfoxide (DMSO) and *N*-Methyl-2-pyrrolidone (NMP)) is demonstrated. It was found that exfoliation using an ultrasonic device with a titanium probe in both solvents produced Cr_2_S_3_ nanosheets with lateral dimensions ranging from 40 to 200 nm and thicknesses of about 10–15 nm (∼6–10 unit cells). Experiments have shown that under liquid-phase exfoliation (LPE) conditions, partial degradation and oxidation of solvents are observed, which has a significant effect on the exfoliation of chromium sulfide. In particular, it leads to partial hydrolysis and oxidation of 2D Cr_2_S_3_, as well as adsorption of solvent degradation and polymerization products on its surface, and affects the properties of the obtained material. These observations seem to be important in view of the further use of NMP and DMSO for the exfoliation of bulk nonlayered van der Waals crystals by LPE. A new understanding of the exfoliation process of non-van der Waals compounds based on the chemical interaction between the dispersion medium and the dispersed phase is proposed.

## Introduction

Two-dimensional (2D) materials have garnered significant interest due to their unique electrical,^1^ optical,^2^ mechanical,^3^ and magnetic properties,^4^ making them pivotal in the development of optoelectronic^5^ and spintronic devices.^6−14^ Among the methods for fabricating these versatile 2D materials, mechanical exfoliation^15−17^ is prominent but not scalable. For widespread applications, a scalable synthesis method is crucial, and here, liquid-phase exfoliation (LPE)^18^ emerges as a well-developed technique.

A key phenomenon in LPE is cavitation,^19−21^ where ultrasonic waves in a liquid create compression and rarefaction cycles, forming vapor bubbles that collapse under high pressure. This collapse generates shock waves, microjets, turbulence, shear forces, and rapid increases in the temperature (up to 5000 K) and pressure (up to 2000 atm). Near solid particles, these effects produce vortex flows and break bonds, aiding in crystal exfoliation.^22−24^ Shock waves create shear forces, sliding layers apart, and exfoliating materials into thin or single layers. This process has allowed for effective exfoliation of two-dimensional nanosheets from layered crystals, like graphite, h-BN, and various layered MQ_2_ compounds (M = Mo, W, Nb, Ta, Ti, Zr, Hf; Q = S, Se),^25^ resulting in their quasi-stable suspensions. The high energy and mechanical forces associated with the bubble collapse can also break the chemical bonds within the structure, overcoming the bonding forces holding the layers together and therefore leading to exfoliation of nonlayered solids.

A new direction in the study of two-dimensional systems began when atomically thin sheets of covalently bonded-only compounds, those without van der Waals bonds, were obtained experimentally by LPE. With the assistance of LPE, 2D transition metal carbides, nitrides, and carbon nitrides called MXenes^26^ were exfoliated from parent MAX phases—a class of laminated materials composed of an early transition metal (M), an A-group element (A), and C, N, B and/or P (X). MXenes have received significant attention and further developed for applications in optoelectronics, photonics, catalysis, and many other areas.^27,28^

The second wave of great interest in such non-van der Waals (non-vdW) 2D materials came when hematene was obtained from hematite (α-Fe_2_O_3_)^29^ and ilmenene was synthesized from ilmenite (FeTiO_3_).^30^ Subsequently, other systems have been obtained from iron(II) chromite (FeCr_2_O_4_),^31^ pyrite (FeS_2_),^32^ iron(III) fluoride,^33^ and WO_3_.^34^ Researchers are particularly interested in these materials because, unlike MXenes where the A layer should be preliminarily removed from the MAX phase by chemical pretreating, their 2D counterparts can be exfoliated directly from bulk materials by LPE without requiring additional procedures.

These materials are compelling due to their new and potentially useful properties, distinct from those of van der Waals-bonded materials.^35,36^ The abundance of dangling bonds increases their chemical activity, making them ideal for surface-active applications.

Non-vdW 2D materials also undergo pronounced structural changes compared to their bulk counterparts in order to lower the surface energy, a process known as surface reorganization. Typically, they experience transverse shrinkage to compensate for broken bonds, with outer atoms moving toward the wafer center, reducing thickness. Additionally, the surface reacts with the environment. For instance, water molecules and dissolved O_2_ can be adsorbed on the surface of nanoparticles. Moreover, under the influence of ultrasound, solvent molecules can degrade and oxidize,^37−39^ leading to the adsorption of degradation products on the surface of nanosheets. Consequently, various functional groups like carboxyl, ketone, and alkyl groups may form on the solid surfaces, reducing nanoparticle aggregation and stabilizing the dispersion while also altering the material properties.^40^

Chromium(III) sulfide has recently attracted increased attention from researchers due to its interesting physical properties. It is theoretically predicted to be a narrow-gap semiconductor with a band gap of 0.45 eV.^41,42^ It has been shown experimentally that the semiconductor properties of 2D Cr_2_S_3_ are determined by the thicknesses of the nanosheets obtained. For example, Cr_2_S_3_ nanosheets with a thickness of one unit cell are p-type semiconductors, while increasing the thickness of the sheets leads to a change in the type of conductivity to n-type.^43,44^ Two-dimensional (2D) Cr_2_S_3_ exhibits significant potential for use in electronics and optoelectronics, particularly in the development of sensitive photodetectors.^42^ Its magnetic properties are also of interest. 2D Cr_2_S_3_ is identified as a ferrimagnetic semiconductor with a monoclinic crystal structure akin to that of NiAs, possessing a Neel temperature of approximately 120 K.^45,46^ Recent research indicates that ultrathin Cr_2_S_3_ nanocrystals feature ultralow switching energy and easily manipulable spins, paving the way for their use in spintronic devices.^47−49^

Presently, the production of ultrathin Cr_2_S_3_ films primarily employs vapor deposition techniques (bottom-up methods).^43,44,50,51^ However, this process is both labor-intensive and costly, making the synthesis of 2D Cr_2_S_3_ crystals a considerable challenge. Consequently, there is a growing need to find more accessible methods for synthesizing 2D magnetic materials.

In this study, we report successful liquid exfoliation of Cr_2_S_3_ in dimethyl sulfoxide and methyl pyrrolidone conducted in an open atmosphere at room temperature. It is important to note that the liquid exfoliation of thermodynamically unstable chromium sulfide may lead to alterations in the composition and properties of the resultant low-dimensional material. This is primarily due to the changes that occur in the dispersion medium under the influence of ultrasonication. Therefore, our work also concentrates on examining the impact of ultrasound on the properties of the solvents and their interaction with the solid phase.

## Results and Discussion

### Characterization of Bulk Chromium Sulfide

**Figure 1:**
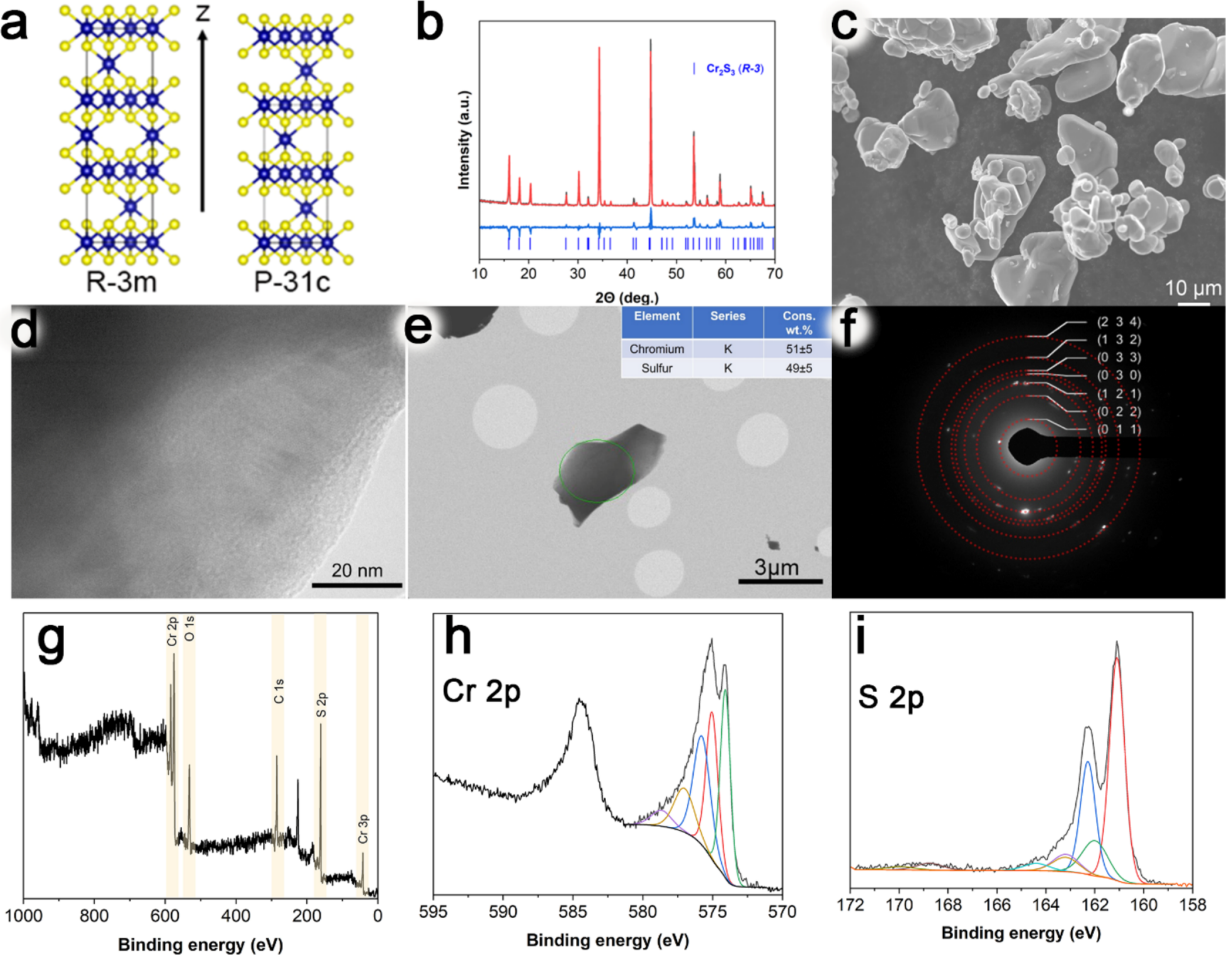
(a) Unit cells of rhombohedral (*R*3̅*m*) and trigonal (*P*3̅1*c*) Cr_2_S_3_. Morphological characterizations and physical properties of commercial bulk Cr_2_S_3_: (b) X-ray diffraction pattern; (c) SEM image; (d) enlarged TEM imaging of one corner of the single particle; (e) TEM image of the single particle and insert is the weight percent of chromium and sulfur per the particle; (f) SAED pattern; (g) full-scan XPS spectrum of bulk Cr_2_S_3_ and the respective high-resolution spectra for (h) Cr 2p and (i) S 2p.

Chromium sulfide (Cr_2_S_3_) exhibits two distinct structural modifications: rhombohedral (*R*3̅) and trigonal (*P*3̅1*c*), as illustrated in Figure 1a. According to the existing literature,^52^ the trigonal variant forms at elevated temperatures, specifically above 800 °C. In contrast, the rhombohedral form represents the more stable configuration of Cr_2_S_3_, maintaining its stability at ambient temperatures. The rhombohedral structure is characterized by densely packed layers of CrS_6_ octahedra that are interconnected via chromium atoms. These structures display a pseudolayered arrangement, predominantly consisting of edge-sharing CrS_6_ octahedra, conforming to the stoichiometry CrS_2_. In this arrangement, approximately 30% of the octahedral interstices in the [CrS_2_]_*x*_^–^ slabs are occupied by Cr^3+^ ions, while the remaining 70% are unoccupied. Occasionally, this material is referred to as Cr_1/3_CrS_2_. This nomenclature underscores the proportion of chromium atoms within the octahedral layers relative to those inserted between these layers. Owing to the significantly lower number of interlayer bonds compared to intralayer bonds, this material has the potential for the development of low-dimensional structures, which shows that the obtained nanoparticles have a narrow particle size distribution and a size range of 10–20 μm.

The results of the crystal structure characterization are illustrated in Figure 1. The narrow and sharp peaks observed in the XRD diffractogram (Figure 1b) indicate the high crystallinity of the material, corresponding to a single phase of Cr_2_S_3_ (JCPDF No. 95-4651, *R*3̅, *a* = 5.9359 ± 0.0003 Å; *c* = 16.6616 ± 0.0001 Å; *V* = 508.41 ± 0.05 Å). The coherent scattering domain size, determined by using Rietveld refinement, is approximately 345 ± 16 nm. It is important to note that the Scherrer equation does not accurately reflect the true sizes of these domains when they exceed 100 nm. According to SEM data (Figure 1c) and TEM data (Figures 1 d,e), the initial sample contains particles of irregular shape and a wide range of sizes (from 2 to 30 μm). The results from electron microdiffraction (Figure 1f) confirm the presence of a chromium sulfide phase. Additionally, scanning transmission electron microscopy (STEM) analysis of a single particle (Figure 1e) reveals that the weight ratios of chromium to sulfur are close to the stoichiometric values for Cr_2_S_3_. The spectra of the bulk Cr_2_S_3_ (Figure 1g–i) are close to those in the literature.^53^ The Cr 2p_3/2_ spectrum (Figure 1g) is conventionally fitted using the multiplet structure similar to that in chromium sulfide (minor amounts of chromium(III) oxide are also observed). The S 2p bands (Figure 1h) can be fitted with the three doublets at S 2p_3/2_ binding energies of 162.6, 163.5, and 161.5 eV representing polysulfide species and a share of monosulfide ions. In addition, there is a weak SO_4_^–^ signal due to surface oxidation (168.5 eV).

### Liquid-Phase Exfoliation

Despite the abundance of studies exploring the impact of the dispersion medium nature on the efficiency of exfoliating van der Waals materials, researchers have yet to reach a consensus on this issue. Various theories are proposed in literature^54−57^ to explain observed patterns and predict successful dispersion media. Most studies suggest using solvents whose surface energy is close to the specific surface tension of the forming nanosheets. This approach reduces the cleavage energy (mixing enthalpy) and facilitates dispersion formation. Since the surface energies of many layered van der Waals compounds, including chemically diverse materials like graphene, hexagonal boron nitride, or molybdenum and tungsten dichalcogenides, are similar and around 40 mJ/m^2^,^25,58^ solvents such as dimethylformamide (DMF), dimethyl sulfoxide (DMSO), and *N*-Methyl-2-pyrrolidone (NMP)^55,59^ can be used for their exfoliation. These solvents have been widely employed for dispersing a range of carbon nanomaterials, including fullerenes, nanotubes, graphene, as well as molybdenum disulfide (MoS_2_) and black phosphorus, resulting in fairly concentrated dispersions of these compounds.^60−65^ However, there are serious doubts that the effectiveness of the solvent in LPE is determined solely by the closeness of the surface energy and specific surface tension values. Jawaid et al.^38^ suggested that the chemical interaction between the dispersion medium and the dispersed phase significantly affects exfoliation efficiency. Furthermore, the surface energy of covalent crystals is much higher than the surface tension of any solvent. Thus, the choice of dispersion medium for liquid exfoliation was made based on literature analysis and preliminary experiments, which showed the best efficiency with NMP and DMSO.

To exfoliate thin chromium sulfide nanoparticles (NPs), we used an ultrasonic bath “Sapphire” for 72 h in a *N*-Methyl-2-pyrrolidone environment (denoted as NMP-NPs-b). Additionally, an ultrasonic device “Volna” with a titanium probe was employed for LPE. In this case, the process was conducted for 20 h in dimethyl sulfoxide (denoted as DMSO-NPs-p) and *N*-methyl-2-pyrrolidone (denoted as NMP-NPs-p). We utilized devices with different power levels (400 and 50 W), resulting in different sonication times.

**Figure 2:**
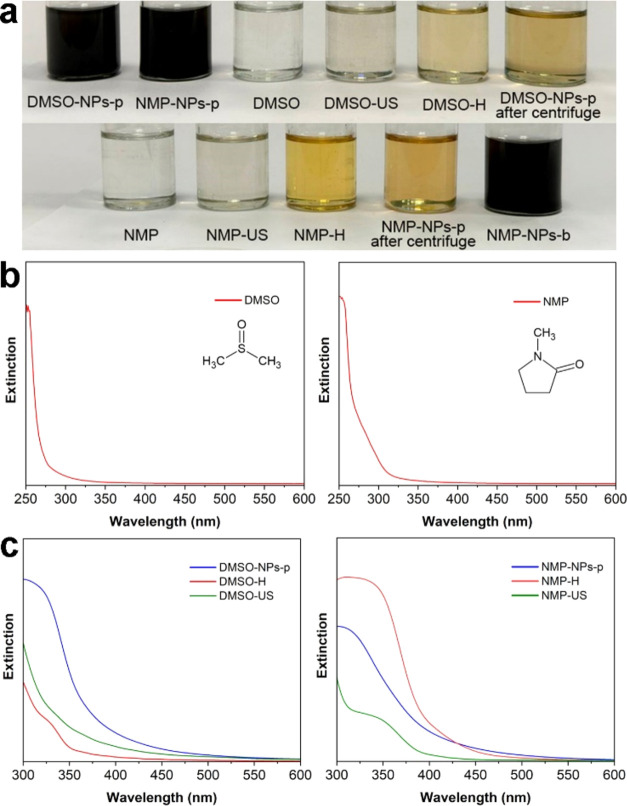
Change in the color of DMSO and NMP after treatment (a): the ultrasound liquid exfoliation of Cr_2_S_3_ during 20 h (DMSO-NPs-p, NMP-NPs-p) and 72 h (NMP-NPs-b), heating to 90 °C (NMP-H, DMSO-H) and ultrasound treatment during 2 h (DMSO-US, NMP-US). UV–Vis– spectra of solvents: (b) before (recorded with empty cuvette as a reference) and (c) after LPE, heating and ultrasound treatment (recorded with pure DMSO or NMP as a reference).

Dispersions with a saturated dark-gray color were obtained (Figure 2a), indirectly confirming exfoliation.^32,38,66^ After separating the larger particles through centrifugation at 9000 rpm, yellow supernatants containing exfoliated particles were obtained. The original solvents exhibit significant absorption in the 200–300 nm range (Figure 2b (spectra recorded relative to air)), while spectra of the supernatants absorb mostly in the range of 300–600 nm, where the absorption of the solvents is minimal (Figure 2c). Furthermore, additional investigation was conducted to account for changes in the solvents during ultrasonic treatment Figure 2c demonstrates spectra of the solvents subjected to 2 h of ultrasonic treatment using the “Volna” device (denoted as DMSO-US, NMP-US; ‘US’ stands for “ultrasonic”) and the solvents after thermal treatment at 90 °C for the same duration (DMSO-H, NMP-H; ‘H’ stands for ‘heating’).

Following two hours of either ultrasonic or thermal treatment of the solvents, a notable change in the color of the samples to bright yellow was observed (Figure 2a), indicating significant chemical alterations. The UV–vis spectra of these solvents exhibit absorption in the 300–450 nm range (Figure 2c), which is characteristic of the degradation products of the solvents. It is well-documented that both DMSO and, particularly, NMP possess complex chemistries, making them susceptible to oxidation and polymerization under sonochemical processing conditions. Yau et al.^37^ highlighted that ultrasonic treatment of NMP can lead to the formation of a wide array of complex byproducts that are difficult to characterize. These byproducts may adsorb onto the surface of solid particles, potentially influencing the properties of the exfoliated products. Notably, since the degradation products absorb light in the 300–450 nm range, absorption spectra in this region are not suitable for determining the optical properties of the resultant 2D materials. However, this limitation is often overlooked in numerous studies.^67−69^

**Figure 3:**
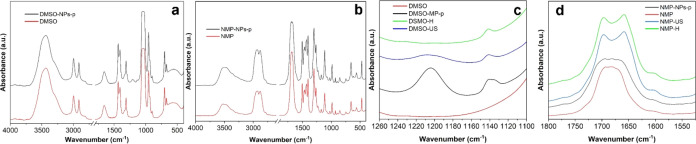
FTIR-spectra of solvents: (a, c) DMSO and (b, d) NMP before (red line) and after (black line) LPE of Cr_2_S_3_, heating (green line) and ultrasound treatment (blue line) in the spectral region of 4000–500 cm^–1^ (a, b) and in the regions of 1260–1100 cm^–1^ (c) and 1800–1500 cm^–1^ (d)).

To accurately capture the changes occurring in the solvents used during LPE, we conducted Fourier-transform infrared (FTIR) spectroscopy was conducted. This analysis was applied to the original solvents that were exposed to ultrasonic and thermal treatments (Figure 3). According to FTIR spectroscopy data, the spectra of the original solvents (DMSO, NMP) and the supernatants postexfoliation (DMSO-NPs-p, NMP-NPs-p) are largely similar (Figure 3a,c). In all spectra, valence vibrations ν(O–H) at 3435 cm^–1^ and δ(H–O–H) vibrations at 1654 cm^–1^, which originate from impurity water, are observed. Vibrations in the regions of 2912 and 2996 cm^–1^ can be attributed to symmetric and asymmetric C–H bonds. Peaks at 1438 and 1405 cm^–1^ correspond to antisymmetric vibrations of CH_3_ groups (δ_as_CH_3_). In the DMSO spectrum, there is also a peak at 1310 cm^–1^, which can be attributed to symmetric vibrations of CH_3_ groups (δ_s_CH_3_) connected to the S atom. The maximum at 1043 cm^–1^ relates to S=O bond vibrations. The peak at 954 cm^–1^ can be associated with C–H vibrations. Additionally, the spectra reveal antisymmetric C–S–C vibrations at 701 cm^–1^. The NMP spectrum features a prominent peak at 1298 cm^–1^, corresponding to the valence vibration of the C–N bond. The carbonyl stretching region (1683 cm^–1^) is observed both in the original NMP and in the supernatant obtained after the exfoliation of Cr_2_S_3_ in the NMP (NMP-NPs-p). Upon closer examination, the following differences can be discerned in the spectra of the solvents before and after the exfoliation process. In the case of DMSO, slight variations are observed in the range 1100–1250 cm^–1^. In the spectrum of DMSO-NPs-p (Figure 3b), weak peaks with maxima at 1134 and 1212 cm^–1^ emerge, which are assigned to antisymmetric vibrations (O=S=O)^70^ and stretching vibrations of the sulfonic acid group,^71^ respectively.

**Scheme 1:**
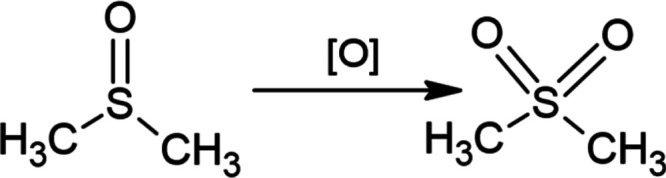
Proposed Scheme for the Decomposition of DMSO

Such changes are likely associated with the oxidation of DMSO into dimethyl sulfone (Scheme 1). This change could be caused by both ultrasonic treatment and heating of the solvent. Weak bands with maxima at 1134 and 1212 cm^–1^ are also observed in the spectra of DMSO-H and DMSO-US samples. In addition, the presence of dimethyl sulfone can also be detected by an NMR examination (Figure S3). After ultrasonic treatment, the same signals are visible in the samples but at 2.92 ppm ^1^H and 41.69 ppm ^13^C. These signals can be attributed to dimethyl sulfone.

**Scheme 2:**
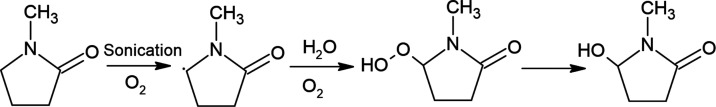
Proposed Scheme for the Oxidation of NMP

**Scheme 3:**
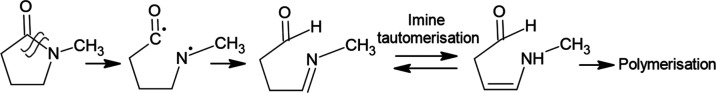
Proposed Scheme for the Decomposition and Polymerization of NMP via Free Radical-Promoted Autoxidation

In the case of NMP, the intensity of the absorption band in the 3400–3500 cm^–1^ range noticeably increases after exfoliation. This enhancement could be attributed to the oxidation of the solvent under the influence of ultrasound, particularly in the presence of dissolved oxygen and impurity water (Scheme 2),^38^ but also to the degradation (Scheme 3).^37^

The appearance of a possible N–H bond and the shift in the carbonyl stretch, in the FTIR data, are consistent with this suggestion^37^ that N–H bond vibrations are in the range of 3400–3500 cm^–1^. The increased absorption intensity in this region is likely due to the opening of the solvent cycle caused by ultrasonic treatment. Additionally, in the FTIR spectra of *N*-Methyl-2-pyrrolidone (NMP), both temperature and ultrasound treatment result in a significant splitting (around 37 cm^–1^) of the carbonyl group peak ν_as,s_(C=O) into symmetric and asymmetric components (Figure 3d). This splitting of the band is due to simultaneous intra- and intermolecular resonance of the carbonyl groups. In this case, the splitting is likely associated with the polymerization of NMP. Additionally, the results of the NMR analysis indicate that the primary degradation product of *N*-methyl-2-pyrrolidone following ultrasound treatment is 4-methylaminobut-3-enoic acid.

Thus, the degradation of the used solvents is caused by both the action of ultrasound and heating. In the case of DMSO, oxidation to dimethyl sulfone is primarily observed. For NMP, in addition to the oxidation process, the opening of the cycle and polymerization of the solvent also take place. The hygroscopic nature of NMP and the extreme local conditions created during ultrasonic treatment of the solvent, including high local temperature associated with cavitation bubbles and active mixing with the atmosphere, create ideal conditions for such transformations in NMP. The oxidation of NMP enhances the efficiency of exfoliation as the resulting reactive *N*- and ω-hydroperoxides oxidize the surface layer of MoS_2_, leading to its delamination without aggressive mechanical processing.^38^ Furthermore, the oxidation products of the solvent, as well as its polymerization, adsorb on the surface of the exfoliated particles, reducing their surface energy and increasing surface charge, which contributes to the stabilization of the forming nanolayers. Such significant changes in the properties of the solid phase under the influence of chemical changes in the dispersion medium are extremely important in the obtained low-dimensional materials and should be taken into account.

**Figure 4:**
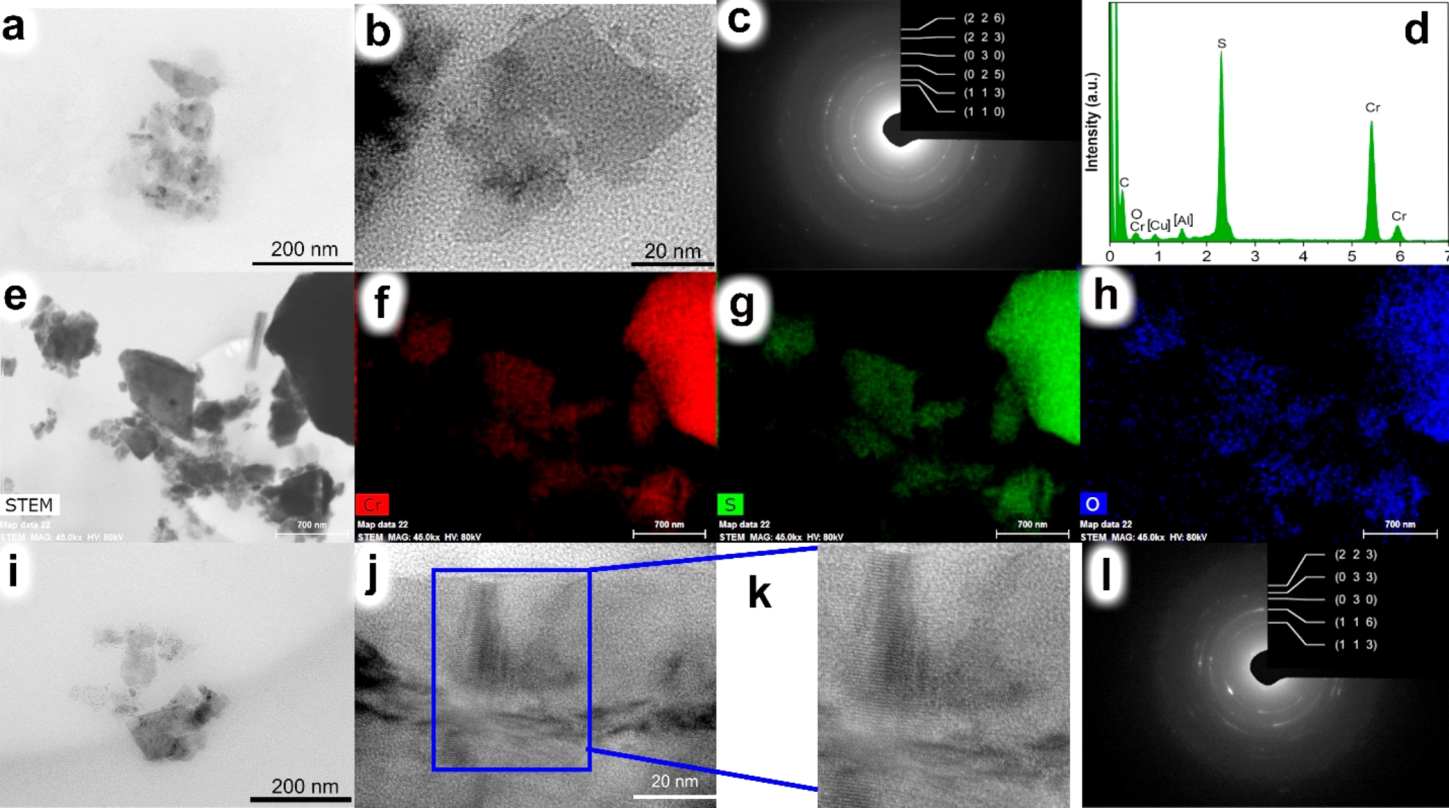
Morphological characterizations and the composition of as-exfoliated NPs Cr_2_S_3_ (NMP-NPs-b and NMP-NPs-p samples): (a, b) TEM images of NMP-NPs-b; (c) SAED pattern of NMP-NPs-b resolves multiple crystallographic planes that correspond to Cr_2_S_3_; (d) energy-dispersive X-ray spectrum of NMP-NPs-b (Al and Cu signals were put in brackets since their source is the sample holder for EDX analysis); (e-h) STEM image and elements (Cr, S and O) mapping image of NMP-NPs-b; (i) TEM image TEM images of NMP-NPs-p; (j) enlarged TEM image of the single particle; (k) enlarged TEM image of the single particle (individual atomic layers are visible); (l) SAED pattern resolving multiple crystallographic planes that correspond to Cr_2_S_3_.

Figure 4 illustrates the systematical characterization of the as-exfoliated Cr_2_S_3_ NPs (NMP-NPs-b). According to the low-magnification TEM image (Figure 4a,b), single Cr_2_S_3_ NPs with lateral size 20–200 nm can be observed (the particle lateral size distribution histogram is presented in Figure S4a). These particles have a light-gray color due to their thickness. Electron microdiffraction results (Figure 4c) confirm the presence of the Cr_2_S_3_ phase in the NMP-NPs-b sample. However, weak oxygen lines in the EDX spectrum (Figure 4d) suggest partial oxidation or hydrolysis of Cr_2_S_3_. Therefore, prolonged (72 h) ultrasonic treatment in NMP in an ultrasonic bath likely leads to the exfoliation of chromium sulfide and the formation of thin nanosheets. However, due to the hygroscopic nature of the solvent and the degradation processes triggered by ultrasonication, partial hydrolysis and oxidation of the obtained 2D material occur. These compositional changes in the material are confirmed by STEM data (Figure 4e–h), where oxygen signals are located in the same spatial region as Cr and S, albeit with lower intensity.

Apart from the partial surface oxidation and hydrolysis of the exfoliated chromium sulfide particles, the presence of oxygen signals in the elemental mapping could also be explained by the adsorption of oxidized solvent intermediates.^38^ X-ray photoelectron spectroscopy of NMP-NPs-b after 20 h (Figure S5) shows peaks for O 1s, C 1s, and Si 2p, with Si likely originating from the reaction vessel material. Despite the clear presence of the Cr_2_S_3_ phase, as confirmed by SAED, the broad scan XPS (Figure S5) does not reveal lines corresponding to Cr and S. The absence of Cr 2p and S 2p lines is due to a thick film of organic contaminants adsorbed on the Cr_2_S_3_ particle surface. Given that XPS is a surface-sensitive technique with a probing depth of 1–10 nm, it can be inferred that the surface film thickness is greater than 10 nm. This film is firmly attached to the NPs and we did not manage to separate it from the flakes during washing with ethyl alcohol, ether, hexane, and other solvents or during continuous vacuum drying.

**Figure 5:**
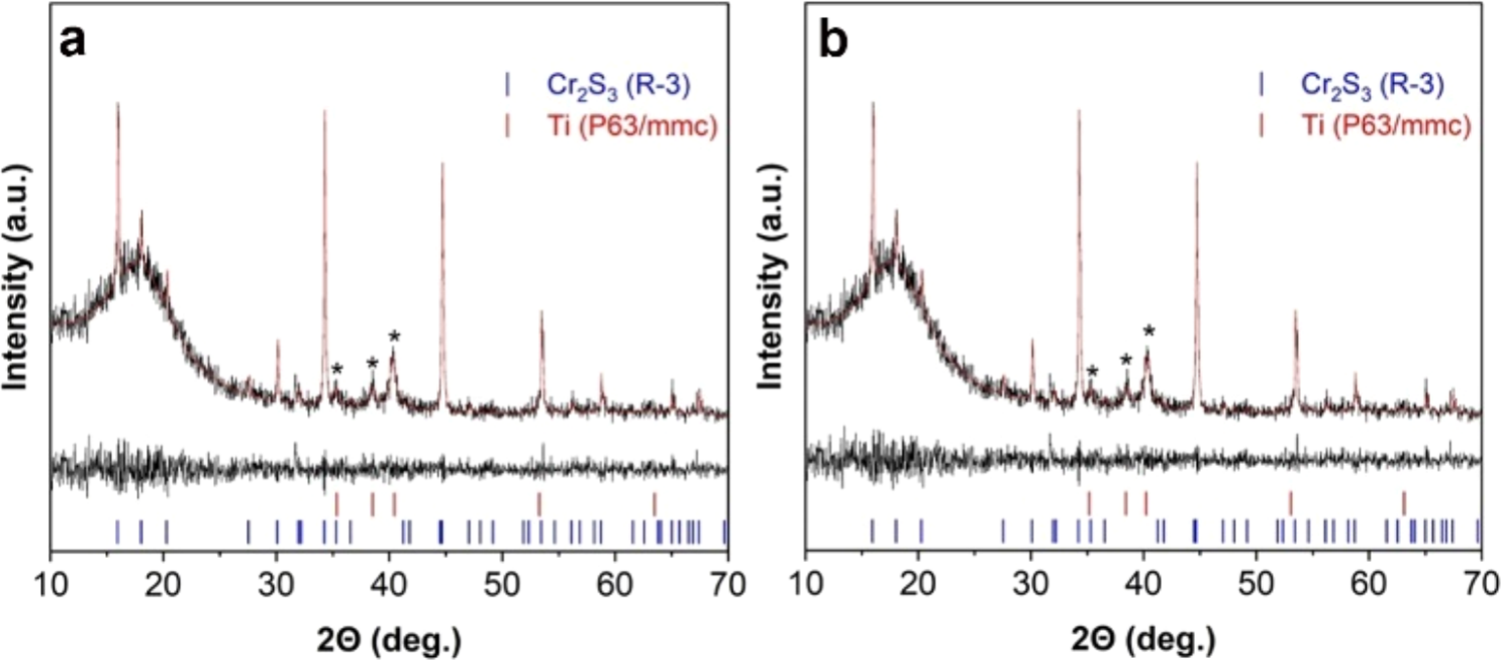
Phase composition of samples DMSO-NPs-p (a) and NMP-NPs-p (b). The red line indicates the calculated model. The difference between both values and the line diagram of phases (Cr_2_S_3_, Ti) are presented in the lower portion of the graph and indicated by the black line.* - Ti (P63/mmc).

Reducing the ultrasonic treatment time to 20 h (NMP-NPs-p) resulted in the formation of Cr_2_S_3_ particles with lateral sizes ranging from 20 to 200 nm (Figure 4i). A particle lateral size distribution histogram is presented in Figure S4b. The obtained particles are light-gray, correlating with their small thickness. TEM data (Figures 4j,k) confirm the presence of monocrystalline Cr_2_S_3_ (FFT pattern is presented in the Supporting Information Figure S8). Electron microdiffraction results (Figure 4l) also confirm the presence of the Cr_2_S_3_ phase. X-ray diffraction data of sediments separated during centrifugation of dispersions obtained by ultrasonic treatment of bulk chromium sulfide in NMP (NMP-NPs-p) and DMSO (DMSO-NPs-p) are shown in Figure 5. The analysis revealed that in addition to the narrow lines characteristic of bulk Cr_2_S_3_ (*R*3̅), the X-ray diagrams of both samples display an amorphous halo in the 2θ 15–20° range. This may be associated with the formation of chromium hydroxide as a result of the partial oxidation and hydrolysis of chromium sulfide. Furthermore, titanium lines (P63/mmc) are present in the X-ray diagrams of the sediments, likely originating from the ultrasonic probe.

**Figure 6:**
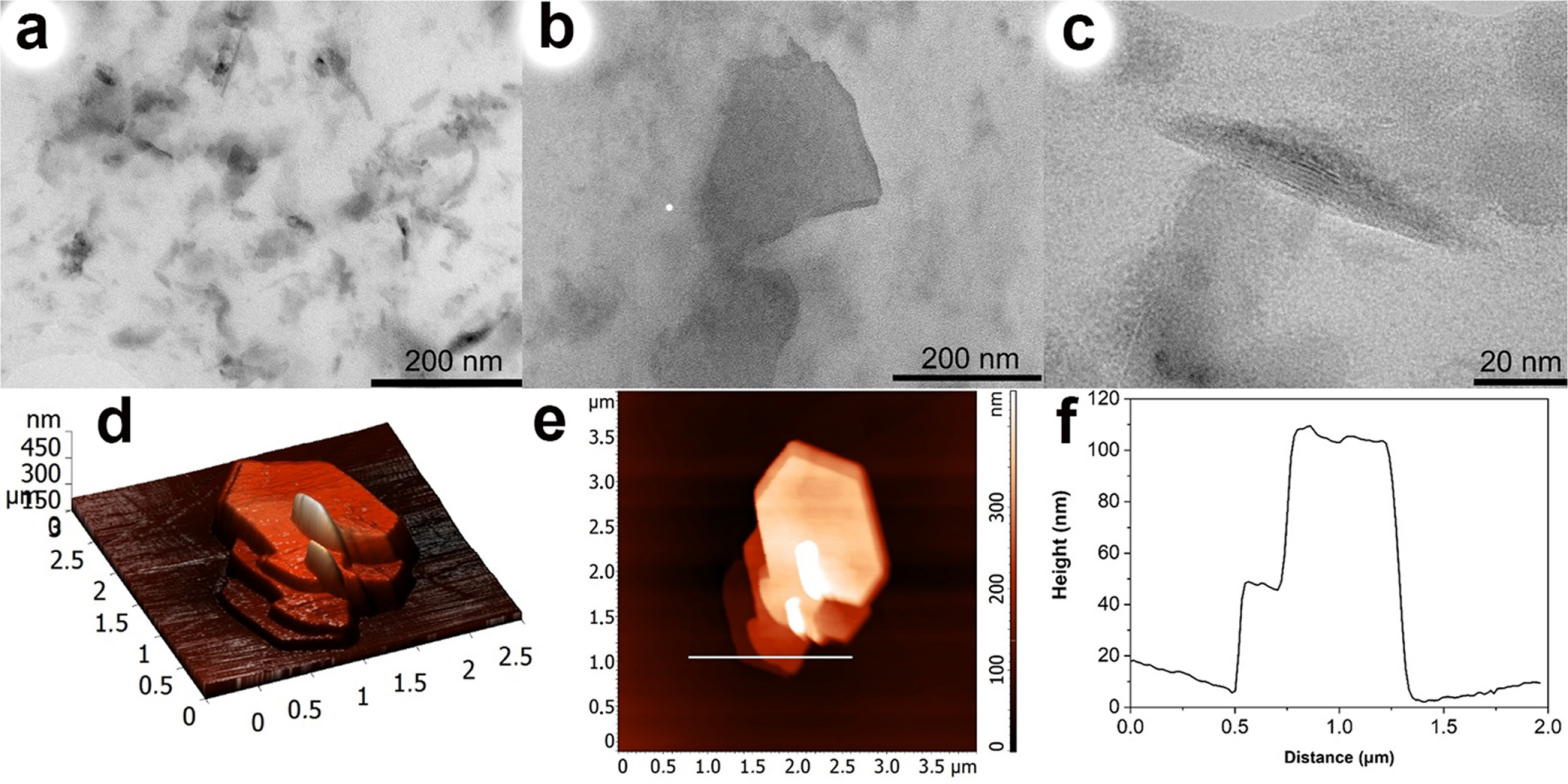
Morphological characterizations and the composition of as-exfoliated Cr_2_S_3_ NPs (sample DMSO-NPs-p): (a) TEM image; (b) TEM images of the single particle after 30.5° tilting of the sample holder; (c) enlarged TEM image of the single particle; (d, e) AFM images. (f) Height profiles along the lines in (e).

Figure 6 shows the results of transmission electron microscopy of the DMSO-NPs-p supernatant separated by centrifugation at 9000 rpm after exfoliation of Cr_2_S_3_ in DMSO. According to the TEM data, Cr_2_S_3_ particles with lateral sizes ranging from 40 to 200 nm were exfoliated. A particle size distribution histogram is presented in Figure S4c. Transmission electron microscopy (TEM) does not provide a clear assessment of the thickness of the obtained particles. However, in Figure 6b, the tilt of the sample holder was adjusted to 30.5°, distinctly revealing the minimal thickness of the observed crystal. Figure 6c likely captures the thickness of one of the particles, which is measured to be ∼10 to 15 nm. Comparing the particle thickness with the lattice parameters of chromium sulfide suggests that the particles consist of 6–10 unit cells. The XPS spectra of the DMSO-NPs-p sample obtained after exfoliation in DMSO are presented in Figure S6. The results show that the surface changes are negligible with respect to the bulk.

For a more detailed determination of the thickness of the obtained particles, atomic force microscopy (AFM) was employed (Figure 6d–f). It is possible to distinctly discern several individual flat particles with lateral dimensions of 100–150 by 200–250 nm, forming stacks that complicate the determination of their true thickness (The diagram of the lateral size distribution and the thickness of sample DMSO-NPs-p is presented in Supporting Information Figure S8). The aggregation of the nanosheets is likely caused by the slow evaporation of dimethyl sulfoxide during sample preparation.^72^ It is also conceivable that the sample in question is not fully peeled but rather exhibits broken edges.

Thus, liquid-phase exfoliation NMP and DMSO results in the formation of particles with similar morphologies, thicknesses, and lateral dimensions. However, exfoliation in NMP is accompanied by the formation of a large number of difficult-to-remove byproducts adsorbed on the surface of the nanoparticles. This poses challenges for analyzing the materials using X-ray Photoelectron Spectroscopy (XPS) and Atomic Force Microscopy (AFM) due to contamination of the cantilever needle. It also alters the surface composition and properties of the nanoparticles. In the case of DMSO, the solvent and its degradation products, formed under the influence of ultrasound, can be effectively removed from the surface of 2D particles by thorough washing with ethyl alcohol. Given that both dispersion media yield a similar product yield, we believe that DMSO, being more stable to the effects of ultrasound and temperature, is a more suitable medium for conducting the LPE process. However, it was not possible to avoid partial oxidation and hydrolysis of chromium sulfide under the experimental conditions when both solvents were used.

## Conclusions

We successfully exfoliated non-van der Waals Cr_2_S_3_ nanocrystals with lateral dimensions ranging from 40 to 200 nm. Electron microdiffraction confirms the presence of the Cr_2_S_3_ phase in the LPE products. However, the EDX spectrum analysis reveals weak oxygen lines, hinting at possible partial oxidation or hydrolysis of Cr_2_S_3_. Our findings also demonstrate that NMP and DMSO, despite being commonly used solvents, pose greater challenges to experimental interpretation than previously anticipated. The results diverge from the conventional theory of LPE efficiency, which relies on matching the surface energies of the solvent and bulk material to achieve low mixing enthalpy. We propose that the key to the LPE efficiency of chromium sulfide in NMP and DMSO lies in the chemical interplay between the dispersion medium and the dispersed phase. FTIR and UV–vis spectroscopy reveal the susceptibility of these solvents to thermal and sonochemical decomposition, as well as polymerization—a characteristic of amides. The resultant oxidation and degradation byproducts adhere to the edges and basal planes of the Cr_2_S_3_ nanocrystals, leading to their oxidation and breaking of covalent bonds. Consequently, alterations in composition and surface properties were observed. According to XPS analysis, the organic surface film exceeds 10 nm in thickness and robustly adheres to the nanosheets. It may remain intact through extensive washing with alcohol, ether, hexane, and other solvents, as well as during prolonged vacuum drying. These findings are particularly significant given the widespread use of NMP and DMSO as dispersion media in LPE. The solvent degradation, caused by traces of water and oxygen, necessitates caution when investigating the properties of the liquid-phase exfoliated 2D materials. Future research on distinguishing the intrinsic properties of the nanosheets influenced by the adsorbed solvent molecules, their degradation, and polymerization products are needed.

Thus, while ultrasonic treatment of bulk chromium sulfide successfully produces thin nanosheets, the hygroscopic nature of the solvents and the presence of dissolved oxygen initiate degradation and oxidation processes. These processes lead to partial hydrolysis and surface contamination of the derived low-dimensional material, affecting its properties and potential applications. This insight is pivotal for the advancement of liquid-phase exfoliation techniques and the development of high-quality 2D materials.

## Experimental Section

### Chemicals

All chemicals were purchased from commercial suppliers. Chromium(III) sulfide (Cr_2_S_3_) (Macklin, China, 99.99%), Dimethyl sulfoxide (DMSO) (Acros Organics, 99.7%); *N*-methyl-2-pyrrolidne (NMP) (Sisco Research Laboratories, 99.5%).

### Preparation of Low-Dimensional Cr_2_S_3_ NPs

Cr_2_S_3_ nanosheets were obtained by the LPE method. Briefly, 100 mg of commercial bulk Cr_2_S_3_ powder was dispersed in 40 mL of DMSO or NMP. Then, the mixture was ultrasonicated with 400 W for 20 h by a device “Volna” with a titanium probe (Center of Ultrasonic Technologies LLC, Biysk Russia) (22 kHz) or with 50 W for 72 h in an ultrasonic bath (Saphir, Russia) at ambient temperature (35 kHz). When using the probe ultrasonic treatment, the solvent vessel was cooled in a 2 L ice bath to prevent the temperature of the sample from rising. During the experiment, the temperature inside the vessel did not exceed 50 °C. After sonication, the mixture was centrifuged for 15 min at 9000 rpm (8241 g) to remove unexfoliated Cr_2_S_3_. The supernatant was collected and analyzed.

### Characterization

The phase composition of samples was determined on a Shimadzu XRD-6000 (Shimadzu Corporation, Kyoto, Japan) diffractometer employing monochromatic Cu Kα radiation. Phase identification was carried out using the PDF-2 database card file. Rietveld refinement of compositions, cell parameter extraction, and sizes of coherent scattering regions (CSR) of obtained materials were carried out in Topas (version 7) software. Transmission electron microscopy (TEM), energy-dispersive X-ray (EDX) analysis, and selected area electron diffraction (SAED) characterization were carried out using a Hitachi 7700 M (Hitachi Corporation, Hitachi, Japan) with accelerating voltage of 110 and 80 kV. Scanning electron microscopy (SEM) analysis was carried out using a JSM-7001F (JEOL Ltd., Tokyo, Japan, using the accelerating voltage of 15 kV). The horizontal attenuated total reflectance Fourier-transform infrared spectroscopy (HATR-FTIR) spectra were recorded on a Tensor 27 (Bruker, Germany) spectrometer equipped with a ZnSe single crystal. The spectra were recorded in the spectral range of 4000–600 cm^–1^ with a resolution of 4 cm^–1^ and 32 scans. The recorded HATR-FTIR spectra were processed using OPUS 7.5 software. Ultraviolet–visible (UV–vis) absorption spectra were collected in a quartz cell with an optical path of 1 cm, employing a GENESYS 10S UV–vis spectrophotometer (Thermo Scientific, Bedford MA) in the range 200–1000 nm. Spectra of solvents after LPE, heating, and ultrasound treatment were recorded with reference spectra collected at pure DMSO and NMP. Pure solvent spectra were recorded using an empty cuvette as a reference. In order to observe the changes that occur in the solvent under the influence of ultrasound and heating at 90 °C, we used a relatively short time period of 2 h, as the temperature of the medium also increased under the ultrasonic treatment.

X-ray photoelectron spectroscopy (XPS) studies were performed using a hydrosol, dried with highly oriented pyrolytic graphite (HOPG) and gently rinsed with water. The spectra were acquired using a SPECS spectrometer (SPECS Gmbh, Berlin, Germany) equipped with a PHOIBOS 150-MCD-9 hemispherical electron analyzer. Spectra were recorded upon excitation with monochromatic radiation of AlKα (*E* = 1486.6 eV). The analyzer pass energy was 10 eV for high-resolution scans and 20 eV for the survey spectra. An electron flood gun was applied to eliminate the inhomogeneous electrostatic charging of the samples; the C 1s peak at 284.45 eV from HOPG was used as a reference. The high-resolution spectra were fitted after the subtraction of Shirley-type background with Gaussian–Lorentzian peak profiles using CasaXPS software (version 2.3.16, Casa Software, Teignmouth, U.K.).

Nuclear magnetic resonance (NMR) spectra were recorded on a Bruker AVANCE 400 spectrometer (Bruker, Germany) using standard 5 mm NMR tubes. ^1^H spectra were acquired using a single pulse at an operating frequency of 600 MHz with a relaxation delay of 5 μs. Water suppression was achieved using a standard zgpr pulse sequence from the Bruker library. ^13^C{^1^H} spectra with proton decoupling were recorded at the operating frequency of 150 MHz with a relaxation delay of 6.5 μs, accumulating 512 scans over 19 h. Chemical shifts were referenced to the signal of tetramethylsilane (TMS) set at 0 391 ppm. All spectra were processed by using the Topspin 3.2 software package.

Atomic force microscopy (AFM) experiments were carried out using a multimode scanning probe microscope Solver P47 (NT-MDT, Moscow, Russia) equipped with a 14 mm scanner having a nominal lateral resolution of about 0.1 nm. The samples were scanned at room temperature in the air using silicon rectangular cantilevers (NSG30, NT-MDT, Moscow) with a typical resonance frequency of about 330 kHz, a stiffness constant of about 40 N/m, and a radius of curvature of the needle of less than 10 nm. Scanning was performed at at least 3–4 points in several regions. The scanning rate was 1.74 Hz. The resolution of the resulting image was 256 × 256 pixels. As a rule, no smoothing or other processing of images was performed except for subtraction of the second-order surface. The surface roughness was calculated from the cross-sectional profile using Nova 026 software.

## Supporting Information Available

NMR spectra; lateral size distribution of samples; XPS spectra of as-exfoliated NPs Cr_2_S_3_; wide spectra; Cr 2p and S 2p XPS spectra of as-exfoliated NPs Cr_2_S_3_ (sample DMSO-NPs-p); Fast Fourier-transform (FFT) pattern of an individual Cr_2_S_3_ nanoparticle; the diagram of the distribution of lateral sizes and the thickness of DMSO-NPs-p (PDF)
