# Supplementary material for: Challenges in Liquid-Phase Exfoliation of Non-van der Waals Cr2S3
Source: ACS Omega. 2024 Nov 15;9(47):46762–72. doi: 10.1021/acsomega.4c02452 (PMC11603257; doi:10.1021/acsomega.4c02452)
Supplement: Supplementary file 1 — ao4c02452_si_001.pdf [file ao4c02452_si_001.pdf]

Supporting information for

Challenges in Liquid-Phase Exfoliation of Non-van der Waals Cr_2S_3

Svetlana V. Saikova [a,b]*, *Aleksandr Yu. Pavlikov* [a], *Diana I. Nemkova* [a], *Alexandr S. Samoilo* [a], *Denis V. Karpov* [a,b], *Anton A. Karacharov* [b], *Svetlana N. Novikova* [b], *Timur Yu. Ivanenko* [b], *Mikhail N. Volochaev* [c], *Galina M. Zeer* [d], *Ye Zhang* [e], *Yuri* L. *Mikhlin* [f], *Hans Ågren* [g], *Artem V. Kuklin* [g]*

[a] School of Non-Ferrous Metals and Material Science, Siberian Federal University, 660041 Krasnoyarsk, Russia

[b] Institute of Chemistry and Chemical Technology, Federal Research Center "Krasnoyarsk Science Center of the Siberian Branch of the Russian Academy of Sciences", Akademgorodok, 660036 Krasnoyarsk, Russia

[c] Kirensky Institute of Physics, Federal Research Center "Krasnoyarsk Science Center of the Siberian Branch of the Russian Academy of Sciences", Akademgorodok, 660036 Krasnoyarsk, Russia

[d] Laboratory of Electron Microscopy, Siberian Federal University, Krasnoyarsk 660041, Russia

[e] School of Chemistry and Chemical Engineering, University of South China, Hengyang 421001, China

[f] Department of Chemistry, Bar-Ilan University, Ramat Gan 52900, Israel

[g] Department of Physics and Astronomy, Uppsala University, P.O. Box 516, SE-751 20 Uppsala, Sweden

* artem.icm@gmail.com (Artem V. Kuklin)
* ssai@mail.ru (Svetlana V. Saikova)

1. Nuclear magnetic resonance

Nuclear magnetic resonance (NMR) spectra were recorded on a Bruker AVANCE 400 spectrometer (Bruker, Germany) using standard 5 mm NMR tubes. ^1H spectra were acquired using a single pulse at the operating frequency of 600 MHz with a relaxation delay of 5 μs. Water suppression was achieved using a standard zgpr pulse sequence from the Bruker library.

$^{13}C\{^{1}H\}$ spectra with proton decoupling were recorded at the operating frequency of 150 MHz with a relaxation delay of 6.5 μs, accumulating 512 scans over 19 hours. Chemical shifts were referenced to the signal of tetramethylsilane (TMS) set at 0 391 ppm. All spectra were processed using the Topspin 3.2 software package. The changes in N-methyl-2-pyrrolidone (NMP) and Dimethylsulfoxide (DMSO) during ultrasonic treatment are further confirmed by NMR spectroscopy data.

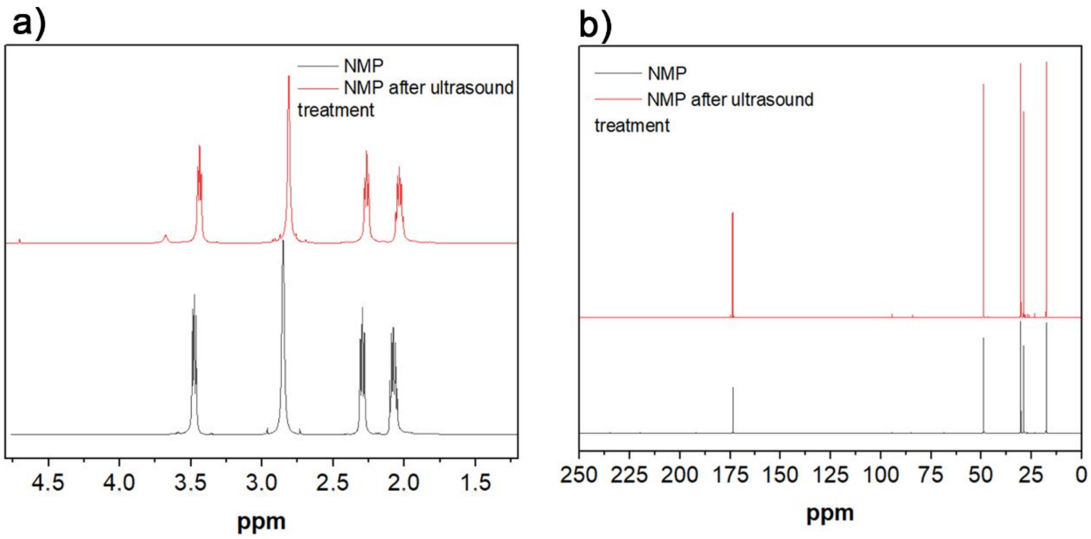

Figure S1. ^{1}H (a) and ^{13}C (b) NMR spectra of NMP before (bottom line) and after (top line) ultrasound treatment

In the 1H NMR spectrum of NMP (Fig. S1 a), four signals are present, one corresponding to the methyl group on the nitrogen atom, and three others to methylene groups of the ring. The signals of CH_2 groups are broadened due to line overlapping arising from the geometric non-equivalence of CH_2 protons and their spin-spin interactions with each other. In the ^{13}C NMR spectrum (Fig. S1 b), three signals of CH_2 groups (17.75, 28.85, 48.87 ppm), a signal of the methyl group at 30.38 ppm, and a signal of C=O located in the deshielded region of the spectrum at 173.74 ppm are observed. The determination of NMP transformation products under ultrasound was conducted using correlation NMR experiments (2D-1H-1H-

DQF-COSY, 2D-1H-13C-HMBC, and 2D-1H-13C-HSQC) (Fig. S2). In the lowfield region, a well -resolved signal characteristic of the carboxyl group proton (11.9 ppm) is observed, which is also identified in the ^{13}C spectrum (174.80 ppm). Signals of 1H and 13C with multiple bonds are located in characteristic regions of the spectra (5-7 ppm for 1H and 70-100 ppm for ^{13}C). The signals of CH_2 and CH_3 groups overlap with signals of the original NMP, however, they can be identified through 2D correlation spectra, from which it follows that the main product of NMP degradation is 4-methylaminobut-3-enoic acid.

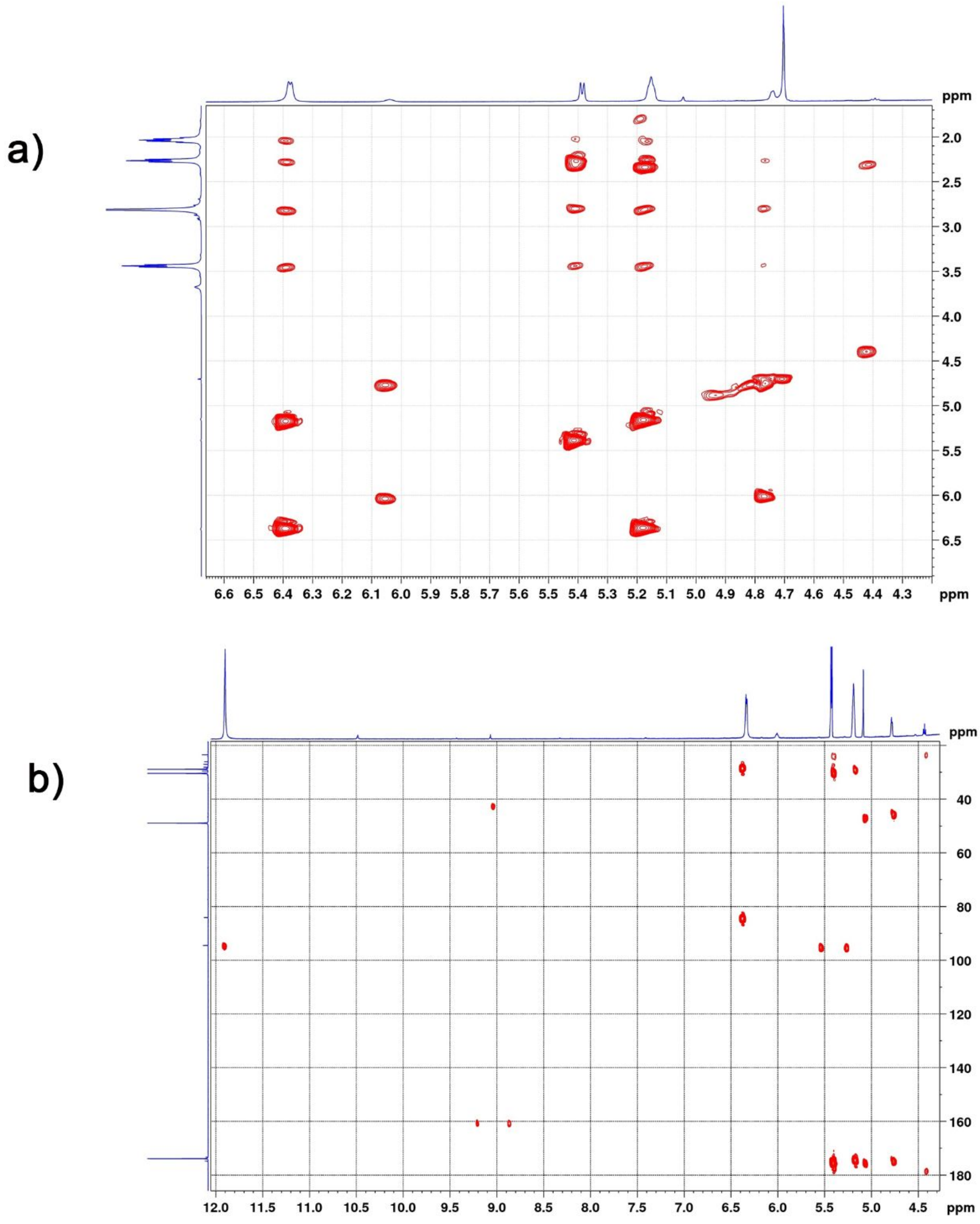

Figure S2. ^{1}H-^{1}H-DQF-COSY (a) и ^{1}H-^{13}C-HMBC (b) NMR spectra for NMP

In the ^{1}H and ^{13}C spectra of DMSO (Figures S3 a and b), signals of magnetically equivalent methyl groups (at 2.50 ppm ^{1}H, 41.25 ppm ^{13}C) are present, as well as trace amounts of water with a characteristic singlet at 3.27 ppm. After ultrasonic treatment, the same signals are visible

in the samples, but at 2.92 ppm ^{1}H and 41.69 ppm ^{13}C. These signals can be attributed to dimethylsulfone.

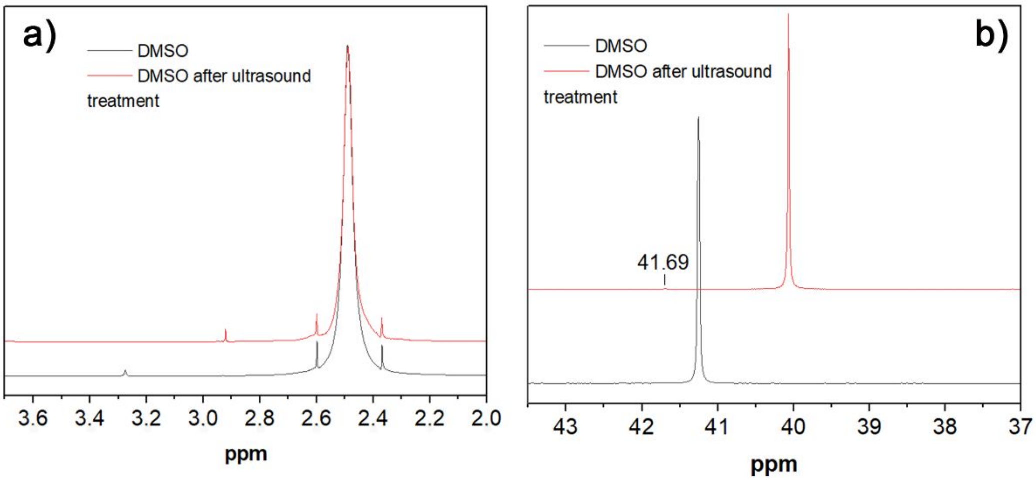

Figure S3. ^{1}H (a) and ^{13}C (b) NMR spectra of DMSO before (bottom line) and after (top line) ultrasound treatment

2. TEM

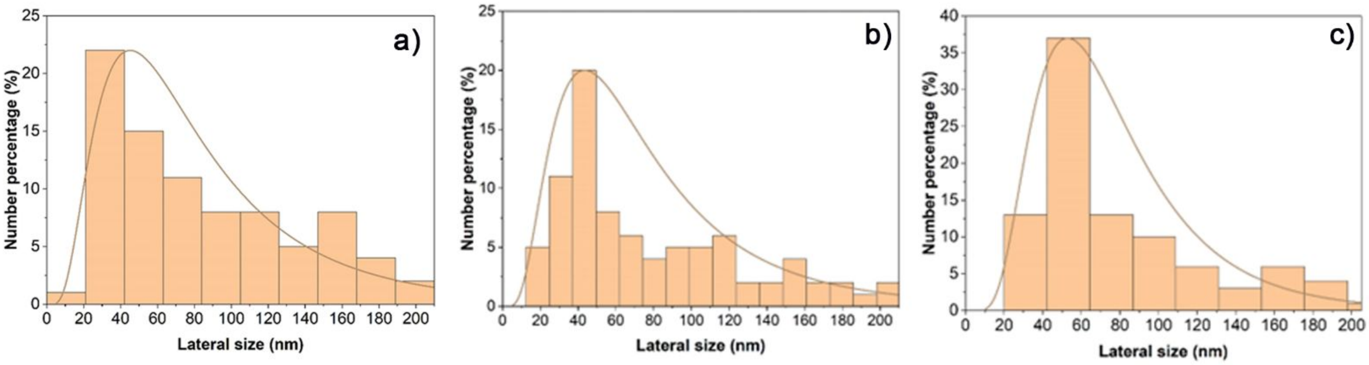

Figure S4. Lateral size distribution of samples NMP-NPs-b (a), NMP-NPs-p (b), DMSO-NPs-p (c)

3. X-Ray photoelectron spectroscopy

(XPS) studies were performed using a hydrosol, dried with highly oriented pyrolytic graphite (HOPG) and gently rinsed with water. The spectra were acquired using a SPECS

spectrometer (SPECS Gmbh, Berlin, Germany) equipped with a PHOIBOS 150-MCD-9 hemispherical electron analyzer. Spectra were recorded upon excitation with a monochromatic radiation of AlKα (E = 1486.6 eV). The analyzer pass energy was 10 eV for high-resolution scans and 20 eV for survey spectra. An electron flood gun was applied to eliminate inhomogeneous electrostatic charging of the samples; the C 1s peak at 284.45 eV from HOPG was used as a reference. The high-resolution spectra were fitted after the subtraction of Shirley-type background with Gaussian–Lorentzian peak profiles using CasaXPS software (version 2.3.16, Casa Software, Teignmouth, UK).

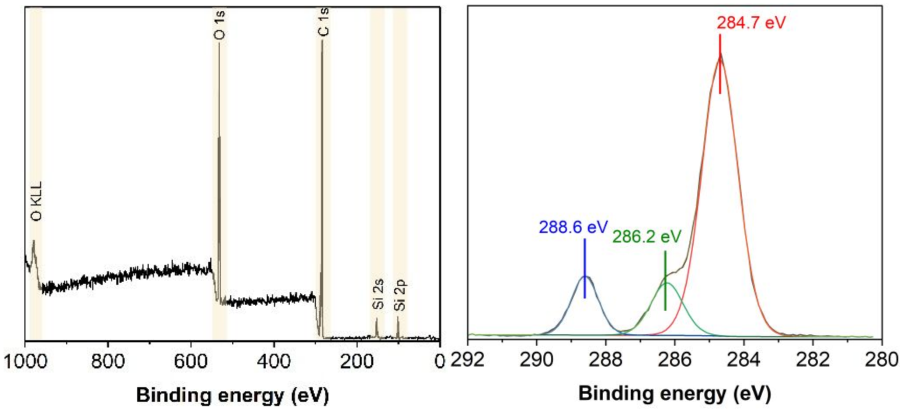

Figure S5. Full scan (left panel) and C1s (right panel) XPS spectra of as-exfoliated NPs Cr_2S_3 (sample NMP-NPs-b)

The broad scan XPS (Figure S5) does not reveal lines corresponding to Cr and S. The C 1s spectral region is complex, displaying 3 constituents dominated by C–C species at 284.7 eV, which derive from sp^3 carbon. The deconvoluted XPS C 1s spectrum also shows peaks at 286.2 eV attributed to the C-OH bond[1] and at 288.6 eV, assigned to the carbonyl or carboxylic group.[2] These are likely the products of the decomposition, oxidation, and polymerization of

methylpyrrolidone adsorbed on the surface of chromium sulfide particles and possibly the flow of slow electrons caused by the acquisition of the XPS spectrum.[3]

After exfoliation in DMSO, all the lines in the spectra of the sample "DMSO-NPs-p" are probably distorted due to inhomogeneous charging. This shows components shifted by about 1 eV to lower binding energies, including C 1s at 283.7 eV, S 2p at 159.8 eV, and Cr 2p3/2 at 573.1 eV.

It is unclear whether this is due to the sample preparation or may arise from, for example, multi- and monolayer entities[4]. Interestingly, Biesinger et al [5]. also reported unusually low BE for S 2p of bulk Cr_2S_3. The higher ratio S/Cr obtained using the Cr 2p band with lower probing depth than that with Cr 3p suggests that the sample surface is enriched in sulfur. The S 2p band is composed of mono-, di- and polysulfide doublets. The signal of sulfate ions at 168.5 eV is very weak, which may be due to their removal from the surface during sample preparation (prolonged washing of the sample with isopropyl alcohol).

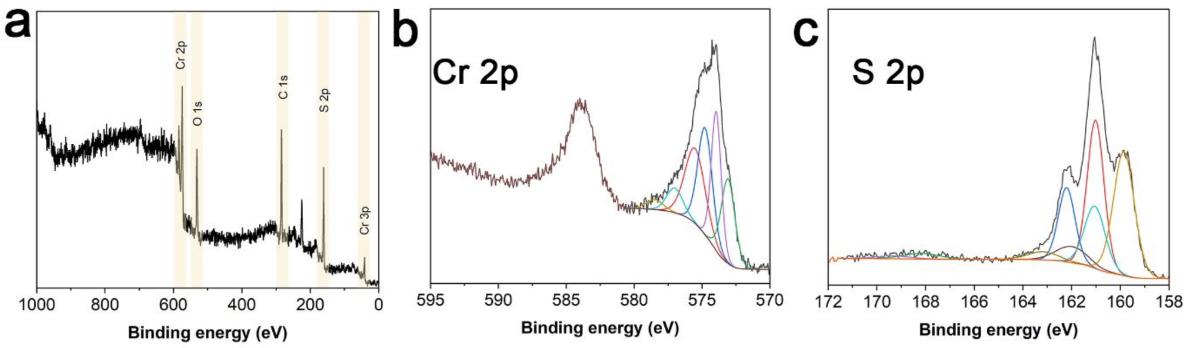

Figure S6. Full scan spectra (a), Cr 2p (b) and S 2p (c) XPS spectra of as-exfoliated NPs Cr_2S_3 (sample DMSO-NPs-p)

4. Fast Fourier transform (FFT)

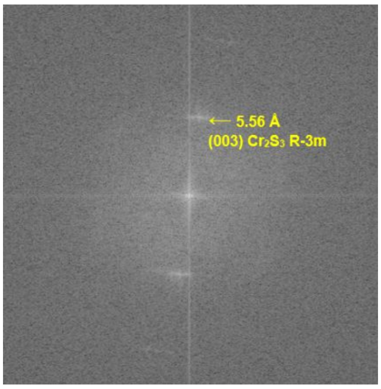

Figure S7. Fast Fourier transform (FFT) pattern of an individual Cr_2S_3 nanoparticle shown in Figure 4k (see main text)

5. Atomic Force Microscopy (AFM)

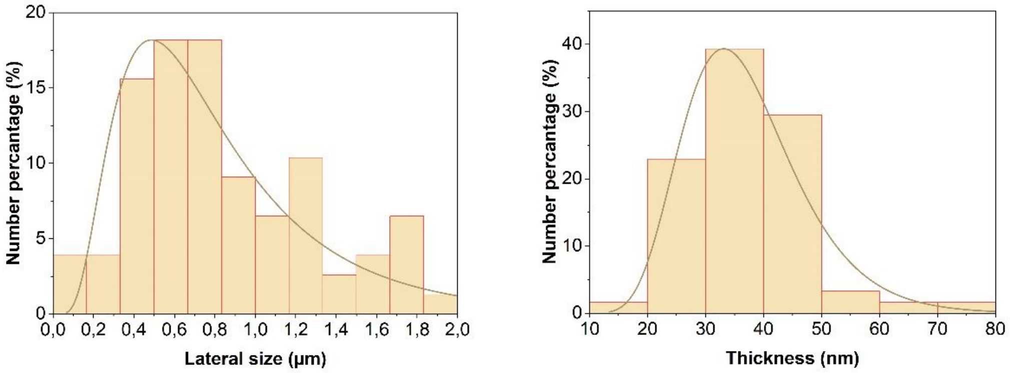

Figure S8. The diagram of the distribution of lateral sizes (left panel) and the thickness(right panel) of DMSO-NPs-p

References

1. Zhang, Z.; Wu, Y. Investigation of the $NaBH_4$-Induced Aggregation of Au Nanoparticles. *Langmuir* **2010**, 26, 9214–9223.

2. Park, J.-W.; Shumaker-Parry, J. S. Strong Resistance of Citrate Anions on Metal Nanoparticles to Desorption under Thiol Functionalization. *ACS Nano* **2015**, 9, 1665–1682.

3. Yau, H. C.; Bayazit, M. K.; Steinke, J. H. G.; Shaffer, M. S. P. Sonochemical degradation of N-methylpyrrolidone and its influence on single-walled carbon nanotube dispersion. *Chemical Communications* **2015**, 51, 16621-16624.
4. Biesinger, M. C., Brown, C., Mycroft, J. R., Davidson, R. D.,McIntyre, N. S.X-ray photoelectron spectroscopy studies of chromium compounds. *Surf. Interface Anal.* **2004**; 36: 1550–1563
5. Su, M., Zhou, W., Jiang, Z., Chen, M., Luo, X., He, J., Yuan, C. Elimination of interlayer potential barriers of chromium sulfide by self-intercalation for enhanced hydrogen evolution reaction. *ACS Applied Materials & Interfaces* **2021**, *13* , 13055-13062.
